# Diphenyl (methyl­amido)­phosphate

**DOI:** 10.1107/S160053681203869X

**Published:** 2012-09-15

**Authors:** Fahimeh Sabbaghi, Mehrdad Pourayoubi, Marek Nečas, Peter Bartoš

**Affiliations:** a Department of Chemistry, Zanjan Branch, Islamic Azad University, Zanjan, Iran; b Department of Chemistry, Ferdowsi University of Mashhad, Mashhad, Iran; c Department of Chemistry, Faculty of Science, Masaryk University, Kotlarska 2, Brno CZ-61137, Czech Republic

## Abstract

The N—H bond in the title compound, C_13_H_14_NO_3_P, is *syn*-oriented relative to the P=O bond. The N atom deviates somewhat from planarity, the sum of the bond angles being 353.3°. The P atom has a distorted tetra­hedral coordination; its bond angles are in the range 93.96 (5)–116.83 (6)°. In the crystal, mol­ecules form centrosymmetric dimers through P=O⋯H—N hydrogen bonds.

## Related literature

For general background, see: Pourayoubi *et al.* (2012 ▶). For bond lengths and angles in a related structure, see: Sabbaghi *et al.* (2011 ▶).
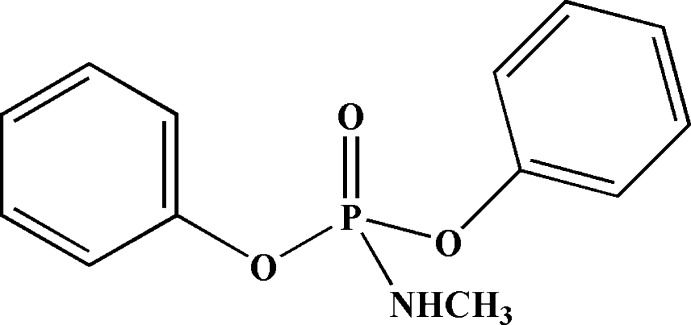


## Experimental

### 

#### Crystal data

C_13_H_14_NO_3_P
*M* *_r_* = 263.22
Monoclinic, 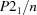

*a* = 9.7652 (5) Å
*b* = 13.6368 (6) Å
*c* = 10.3537 (5) Å
β = 114.217 (6)°
*V* = 1257.43 (12) Å^3^
*Z* = 4
Mo *K*α radiation
μ = 0.22 mm^−1^
*T* = 120 K
0.50 × 0.50 × 0.40 mm

#### Data collection

Oxford Diffraction Xcalibur (Sapphire2) diffractometer
Absorption correction: multi-scan (*CrysAlis RED*; Oxford Diffraction, 2009 ▶) *T*_min_ = 0.939, *T*_max_ = 1.000
14649 measured reflections
2212 independent reflections
1871 reflections with *I* > 2σ(*I*)
*R*_int_ = 0.022

#### Refinement

*R*[*F* ^2^ > 2σ(*F* ^2^)] = 0.028
*wR*(*F* ^2^) = 0.079
*S* = 1.09
2212 reflections
168 parameters
H atoms treated by a mixture of independent and constrained refinement
Δρ_max_ = 0.23 e Å^−3^
Δρ_min_ = −0.29 e Å^−3^

### 

Data collection: *CrysAlis CCD* (Oxford Diffraction, 2009 ▶); cell refinement: *CrysAlis CCD*; data reduction: *CrysAlis RED* (Oxford Diffraction, 2009 ▶); program(s) used to solve structure: *SHELXS97* (Sheldrick, 2008 ▶); program(s) used to refine structure: *SHELXL97* (Sheldrick, 2008 ▶); molecular graphics: *Mercury* (Macrae *et al.*, 2008 ▶); software used to prepare material for publication: *SHELXTL* (Sheldrick, 2008 ▶).

## Supplementary Material

Crystal structure: contains datablock(s) I, global. DOI: 10.1107/S160053681203869X/ld2070sup1.cif

Structure factors: contains datablock(s) I. DOI: 10.1107/S160053681203869X/ld2070Isup2.hkl

Additional supplementary materials:  crystallographic information; 3D view; checkCIF report

## Figures and Tables

**Table 1:** Hydrogen-bond geometry (Å, °)

*D*—H⋯*A*	*D*—H	H⋯*A*	*D*⋯*A*	*D*—H⋯*A*
N1—H1*N*⋯O1^i^	0.788 (18)	2.141 (18)	2.9106 (17)	165.1 (18)

Symmetry code: (i) 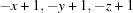.

**Fig. 1.**
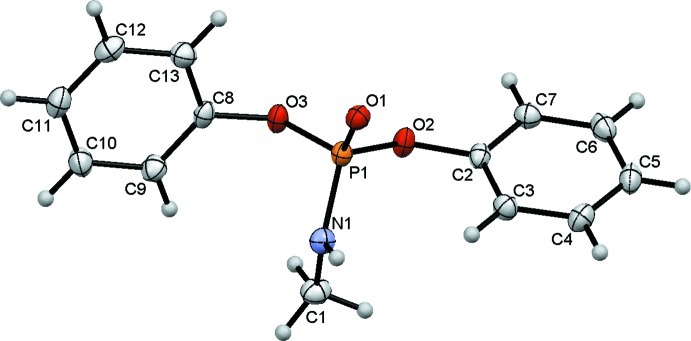
The molecular structure of the title compound with ellipsoids shown at the 50% probability level; H atoms are drawn as small spheres of arbitrary radii.

**Fig. 2.**
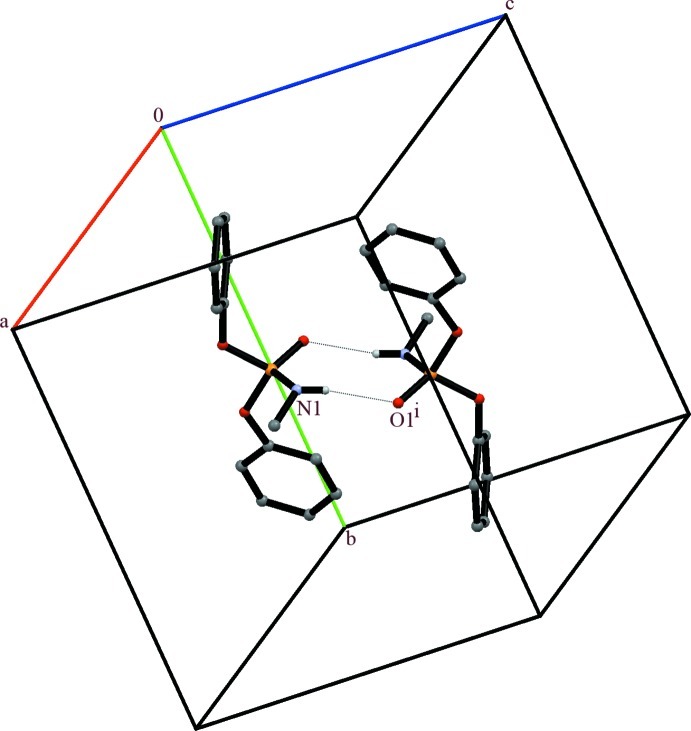
The hydrogen-bonded inversion dimer (pair of P═O···H—N hydrogen bonds is shown by dotted lines) in the crystal structure. The hydrogen atoms not involved in hydrogen bonding have been omitted for clarity.

## supplementary crystallographic information

### Comment

The single-crystal X-ray determination of the title compound, P(O)[OC_6_H_5_]_2_[NHCH_3_] (Fig. 1), was performed due to our interest on the synthesis and structure of phosphorus(V)-nitrogen compounds (Pourayoubi *et al.*, 2012; Sabbaghi *et al.*, 2011).

The P═O (1.4632 (10) Å), P—O (1.5875 (10) and 1.5949 (10) Å), P—N (1.6148 (13) Å) and C—O (1.4037 (18) and 1.4108 (17) Å) bond lengths are within the expected values for analogous compounds with a P(O)(O)_2_(N) skeleton (Sabbaghi *et al.*, 2011).

Angles around phosphorus [O1—P1—O3 116.83 (6)°, O1—P1—O2 114.42 (6)°, O3—P1—O2 93.96 (5)°, O1—P1—N1 112.78 (6)°, O3—P1—N1 106.80 (6)° and O2—P1—N1 110.45 (6)°] are characteristic for a distorted tetrahedral geometry.

The N1 atom deviates from P1C1H1N plane by 0.178 (9) Å.

Also, the sum of valence angles around N1 [353 (1)°] deviates from 360°, as a measure of its pyramidality.

The C—N—P angle (122.53 (10)°) and C—O—P (123.35 (9) and 121.01 (9)°) angles are standard for this family of compounds (Sabbaghi *et al.*, 2011).

In the crystal, pairs of intermolecular P═O···H—N hydrogen bonds form inversion dimers, Table 1 and Fig. 2.

### Experimental

A mixture of [CH_3_NH_3_]Cl (2 mmol) and N(C_2_H_5_)_3_ (4 mmol) in dry CH_3_CN (15 ml) was added to a solution of [C_6_H_5_O]_2_P(O)Cl (2 mmol) in the same solvent (20 ml) on ice bath. After stirring for 4 h, the solvent was removed and the product was washed with distilled water and recrystallized from CH_3_CN/n-C_6_H_14_ at room temperature. The single crystals suitable for X-ray analysis were obtained from this solution after a few days at room temperature.

### Refinement

All carbon-bound H atoms were placed at calculated positions and were refined as riding with their *U*_iso_ set to either 1.2*U*_eq_ or 1.5*U*_eq_ (methyl) of the respective carrier atoms; the methyl H atoms were allowed to rotate about the N—C bond. The nitrogen-bound H atom was located in a difference Fourier map and refined isotropically.

### Crystal data

**Table:**

C_13_H_14_NO_3_P	*F*(000) = 552
*M**_r_* = 263.22	*D*_x_ = 1.390 Mg m^−^^3^
Monoclinic, *P*2_1_/*n*	Mo *K*α radiation, λ = 0.71073 Å
*a* = 9.7652 (5) Å	Cell parameters from 7682 reflections
*b* = 13.6368 (6) Å	θ = 2.8–27.1°
*c* = 10.3537 (5) Å	µ = 0.22 mm^−^^1^
β = 114.217 (6)°	*T* = 120 K
*V* = 1257.43 (12) Å^3^	Block, white
*Z* = 4	0.50 × 0.50 × 0.40 mm

### Data collection

**Table:**

Oxford Diffraction Xcalibur (Sapphire2) diffractometer	2212 independent reflections
Radiation source: Enhance (Mo) X-ray Source	1871 reflections with *I* > 2σ(*I*)
Graphite monochromator	*R*_int_ = 0.022
Detector resolution: 8.4353 pixels mm^-1^	θ_max_ = 25.0°, θ_min_ = 2.8°
ω scan	*h* = −11→11
Absorption correction: multi-scan (*CrysAlis RED*; Oxford Diffraction, 2009)	*k* = −12→16
*T*_min_ = 0.939, *T*_max_ = 1.000	*l* = −12→12
14649 measured reflections	

### Refinement

**Table:**

Refinement on *F*^2^	Primary atom site location: heavy-atom method
Least-squares matrix: full	Secondary atom site location: difference Fourier map
*R*[*F*^2^ > 2σ(*F*^2^)] = 0.028	Hydrogen site location: inferred from neighbouring sites
*wR*(*F*^2^) = 0.079	H atoms treated by a mixture of independent and constrained refinement
*S* = 1.09	*w* = 1/[σ^2^(*F*_o_^2^) + (0.046*P*)^2^ + 0.1667*P*] where *P* = (*F*_o_^2^ + 2*F*_c_^2^)/3
2212 reflections	(Δ/σ)_max_ = 0.001
168 parameters	Δρ_max_ = 0.23 e Å^−^^3^
0 restraints	Δρ_min_ = −0.29 e Å^−^^3^

### Special details

**Table:**

Geometry. All e.s.d.'s (except the e.s.d. in the dihedral angle between two l.s. planes) are estimated using the full covariance matrix. The cell e.s.d.'s are taken into account individually in the estimation of e.s.d.'s in distances, angles and torsion angles; correlations between e.s.d.'s in cell parameters are only used when they are defined by crystal symmetry. An approximate (isotropic) treatment of cell e.s.d.'s is used for estimating e.s.d.'s involving l.s. planes.
Refinement. Refinement of *F*^2^ against ALL reflections. The weighted *R*-factor *wR* and goodness of fit *S* are based on *F*^2^, conventional *R*-factors *R* are based on *F*, with *F* set to zero for negative *F*^2^. The threshold expression of *F*^2^ > σ(*F*^2^) is used only for calculating *R*-factors(gt) *etc*. and is not relevant to the choice of reflections for refinement. *R*-factors based on *F*^2^ are statistically about twice as large as those based on *F*, and *R*- factors based on ALL data will be even larger.

### Fractional atomic coordinates and isotropic or equivalent isotropic displacement parameters (Å^2^)

**Table:**

	*x*	*y*	*z*	*U*_iso_*/*U*_eq_	
P1	0.51390 (4)	0.43348 (3)	0.30989 (4)	0.01769 (14)	
O1	0.38345 (11)	0.43725 (7)	0.34520 (10)	0.0208 (3)	
O2	0.50768 (12)	0.50885 (7)	0.18982 (10)	0.0223 (3)	
O3	0.52841 (11)	0.34002 (7)	0.22495 (10)	0.0211 (3)	
N1	0.67107 (14)	0.44375 (9)	0.44729 (14)	0.0207 (3)	
H1N	0.662 (2)	0.4674 (13)	0.513 (2)	0.032 (5)*	
C1	0.81454 (17)	0.45739 (12)	0.43646 (17)	0.0273 (4)	
H1A	0.8961	0.4325	0.5225	0.041*	
H1B	0.8303	0.5273	0.4256	0.041*	
H1C	0.8133	0.4214	0.3540	0.041*	
C2	0.47748 (15)	0.60922 (11)	0.19327 (15)	0.0188 (3)	
C3	0.51171 (16)	0.66074 (11)	0.31815 (15)	0.0226 (3)	
H3	0.5552	0.6285	0.4072	0.027*	
C4	0.48104 (17)	0.76048 (12)	0.31004 (17)	0.0257 (4)	
H4	0.5030	0.7967	0.3946	0.031*	
C5	0.41887 (17)	0.80803 (12)	0.18051 (17)	0.0267 (4)	
H5	0.3990	0.8764	0.1762	0.032*	
C6	0.38592 (17)	0.75473 (12)	0.05730 (17)	0.0269 (4)	
H6	0.3434	0.7869	−0.0318	0.032*	
C7	0.41444 (16)	0.65494 (11)	0.06295 (15)	0.0228 (3)	
H7	0.3910	0.6185	−0.0217	0.027*	
C8	0.54261 (16)	0.24509 (11)	0.28322 (14)	0.0195 (3)	
C9	0.68401 (17)	0.20402 (12)	0.34803 (16)	0.0249 (4)	
H9	0.7707	0.2406	0.3583	0.030*	
C10	0.69714 (18)	0.10862 (12)	0.39770 (16)	0.0272 (4)	
H10	0.7936	0.0795	0.4431	0.033*	
C11	0.57023 (18)	0.05567 (12)	0.38137 (16)	0.0264 (4)	
H11	0.5797	−0.0099	0.4149	0.032*	
C12	0.42946 (17)	0.09791 (12)	0.31628 (16)	0.0276 (4)	
H12	0.3427	0.0613	0.3056	0.033*	
C13	0.41468 (17)	0.19365 (12)	0.26646 (16)	0.0248 (4)	
H13	0.3184	0.2231	0.2217	0.030*	

### Atomic displacement parameters (Å^2^)

**Table:**

	*U*^11^	*U*^22^	*U*^33^	*U*^12^	*U*^13^	*U*^23^
P1	0.0214 (2)	0.0152 (2)	0.0177 (2)	0.00056 (15)	0.00922 (16)	−0.00031 (15)
O1	0.0221 (5)	0.0185 (6)	0.0227 (5)	−0.0005 (4)	0.0100 (4)	−0.0020 (4)
O2	0.0332 (6)	0.0155 (6)	0.0210 (5)	0.0015 (4)	0.0141 (5)	0.0010 (4)
O3	0.0301 (6)	0.0149 (6)	0.0202 (5)	0.0020 (4)	0.0122 (4)	−0.0002 (4)
N1	0.0217 (7)	0.0224 (8)	0.0202 (7)	0.0009 (5)	0.0109 (6)	−0.0024 (6)
C1	0.0225 (8)	0.0297 (10)	0.0317 (9)	−0.0026 (7)	0.0130 (7)	−0.0037 (7)
C2	0.0188 (7)	0.0150 (8)	0.0248 (8)	−0.0001 (6)	0.0112 (6)	0.0010 (6)
C3	0.0255 (8)	0.0215 (9)	0.0203 (8)	−0.0002 (6)	0.0089 (6)	0.0012 (6)
C4	0.0296 (8)	0.0205 (9)	0.0288 (8)	−0.0019 (7)	0.0138 (7)	−0.0039 (7)
C5	0.0276 (8)	0.0169 (9)	0.0375 (9)	0.0032 (6)	0.0155 (7)	0.0031 (7)
C6	0.0260 (8)	0.0256 (9)	0.0275 (8)	0.0041 (7)	0.0095 (7)	0.0088 (7)
C7	0.0238 (8)	0.0244 (9)	0.0202 (8)	0.0000 (6)	0.0089 (6)	0.0004 (6)
C8	0.0278 (8)	0.0143 (8)	0.0184 (7)	0.0001 (6)	0.0116 (6)	−0.0021 (6)
C9	0.0241 (8)	0.0215 (9)	0.0306 (8)	−0.0022 (6)	0.0127 (7)	0.0000 (7)
C10	0.0273 (9)	0.0221 (9)	0.0318 (9)	0.0053 (7)	0.0117 (7)	0.0036 (7)
C11	0.0380 (9)	0.0184 (9)	0.0254 (8)	−0.0003 (7)	0.0156 (7)	0.0006 (7)
C12	0.0293 (9)	0.0259 (10)	0.0294 (9)	−0.0079 (7)	0.0138 (7)	−0.0029 (7)
C13	0.0220 (8)	0.0253 (10)	0.0255 (8)	0.0002 (7)	0.0083 (6)	−0.0019 (7)

### Geometric parameters (Å, º)

**Table:**

P1—O1	1.4632 (10)	C5—C6	1.387 (2)
P1—O3	1.5875 (10)	C5—H5	0.9500
P1—O2	1.5949 (10)	C6—C7	1.385 (2)
P1—N1	1.6148 (13)	C6—H6	0.9500
O2—C2	1.4037 (18)	C7—H7	0.9500
O3—C8	1.4108 (17)	C8—C13	1.380 (2)
N1—C1	1.4625 (18)	C8—C9	1.382 (2)
N1—H1N	0.788 (18)	C9—C10	1.385 (2)
C1—H1A	0.9800	C9—H9	0.9500
C1—H1B	0.9800	C10—C11	1.383 (2)
C1—H1C	0.9800	C10—H10	0.9500
C2—C7	1.381 (2)	C11—C12	1.384 (2)
C2—C3	1.387 (2)	C11—H11	0.9500
C3—C4	1.388 (2)	C12—C13	1.389 (2)
C3—H3	0.9500	C12—H12	0.9500
C4—C5	1.386 (2)	C13—H13	0.9500
C4—H4	0.9500		
			
O1—P1—O3	116.83 (6)	C4—C5—H5	120.4
O1—P1—O2	114.42 (6)	C6—C5—H5	120.4
O3—P1—O2	93.96 (5)	C7—C6—C5	120.62 (14)
O1—P1—N1	112.78 (6)	C7—C6—H6	119.7
O3—P1—N1	106.80 (6)	C5—C6—H6	119.7
O2—P1—N1	110.45 (6)	C2—C7—C6	119.08 (14)
C2—O2—P1	123.35 (9)	C2—C7—H7	120.5
C8—O3—P1	121.01 (9)	C6—C7—H7	120.5
C1—N1—P1	122.53 (10)	C13—C8—C9	121.84 (14)
C1—N1—H1N	117.4 (13)	C13—C8—O3	119.17 (13)
P1—N1—H1N	113.4 (14)	C9—C8—O3	118.87 (13)
N1—C1—H1A	109.5	C8—C9—C10	118.86 (14)
N1—C1—H1B	109.5	C8—C9—H9	120.6
H1A—C1—H1B	109.5	C10—C9—H9	120.6
N1—C1—H1C	109.5	C11—C10—C9	120.19 (15)
H1A—C1—H1C	109.5	C11—C10—H10	119.9
H1B—C1—H1C	109.5	C9—C10—H10	119.9
C7—C2—C3	121.52 (14)	C10—C11—C12	120.21 (15)
C7—C2—O2	115.43 (13)	C10—C11—H11	119.9
C3—C2—O2	123.03 (13)	C12—C11—H11	119.9
C2—C3—C4	118.46 (14)	C11—C12—C13	120.20 (15)
C2—C3—H3	120.8	C11—C12—H12	119.9
C4—C3—H3	120.8	C13—C12—H12	119.9
C5—C4—C3	121.02 (15)	C8—C13—C12	118.69 (14)
C5—C4—H4	119.5	C8—C13—H13	120.7
C3—C4—H4	119.5	C12—C13—H13	120.7
C4—C5—C6	119.30 (15)		
			
O1—P1—O2—C2	50.74 (12)	C4—C5—C6—C7	0.1 (2)
O3—P1—O2—C2	172.62 (10)	C3—C2—C7—C6	0.5 (2)
N1—P1—O2—C2	−77.81 (11)	O2—C2—C7—C6	−177.80 (12)
O1—P1—O3—C8	−61.57 (11)	C5—C6—C7—C2	−0.6 (2)
O2—P1—O3—C8	178.47 (10)	P1—O3—C8—C13	85.76 (15)
N1—P1—O3—C8	65.73 (11)	P1—O3—C8—C9	−98.12 (14)
O1—P1—N1—C1	−170.21 (11)	C13—C8—C9—C10	−0.2 (2)
O3—P1—N1—C1	60.14 (13)	O3—C8—C9—C10	−176.17 (13)
O2—P1—N1—C1	−40.78 (14)	C8—C9—C10—C11	0.5 (2)
P1—O2—C2—C7	−153.60 (11)	C9—C10—C11—C12	−0.5 (2)
P1—O2—C2—C3	28.15 (18)	C10—C11—C12—C13	0.2 (2)
C7—C2—C3—C4	0.1 (2)	C9—C8—C13—C12	−0.1 (2)
O2—C2—C3—C4	178.21 (13)	O3—C8—C13—C12	175.92 (13)
C2—C3—C4—C5	−0.6 (2)	C11—C12—C13—C8	0.0 (2)
C3—C4—C5—C6	0.5 (2)		

### Hydrogen-bond geometry (Å, º)

**Table:**

*D*—H···*A*	*D*—H	H···*A*	*D*···*A*	*D*—H···*A*
N1—H1*N*···O1^i^	0.788 (18)	2.141 (18)	2.9106 (17)	165.1 (18)

Symmetry code: (i) −*x*+1, −*y*+1, −*z*+1.
